# The role of general psychosocial factors for the use of cancer screening—Findings of a population‐based observational study among older adults in Germany

**DOI:** 10.1002/cam4.1226

**Published:** 2017-10-13

**Authors:** André Hajek, Jens‐Oliver Bock, Hans‐Helmut König

**Affiliations:** ^1^ Department of Health Economics and Health Services Research University Medical Center Hamburg‐Eppendorf Hamburg Germany

**Keywords:** Cancer screening, life satisfaction, psychosocial factors, self‐efficacy, self‐esteem

## Abstract

Within the framework of the health‐belief model, some studies exist investigating the association between *illness‐specific* psychosocial factors and the use of cancer screenings. However, studies investigating the association between *general* psychosocial factors and the use of cancer screenings are missing. Thus, this study aimed at examining the association between well‐established general psychosocial factors and the use of cancer screenings. Data were gathered from a large, population‐based sample of community‐dwelling individuals aged 40 and above in Germany (*n* = 7673; in 2014). Loneliness, cognitive well‐being, affective well‐being (negative and positive affect), optimism, self‐efficacy, self‐esteem, self‐regulation, perceived autonomy, perceived stress, and perceived social exclusion were used as general psychosocial factors. Furthermore, individuals were asked whether they regularly underwent early cancer screening in the past years (yes; no). A total of 65.6% of the individuals used cancer screening. Adjusting for sociodemographic factors, self‐rated health, morbidity and lifestyle factors, multiple logistic regressions revealed that the use of cancer screening is positively associated with decreased loneliness, cognitive well‐being, optimism, self‐efficacy, self‐esteem, self‐regulation, perceived autonomy, decreased perceived stress, decreased perceived social exclusion, and positive affect, while it is not associated with negative affect. This study stresses the strong association between general psychosocial factors and the use of cancer screening. This knowledge might be fruitful to address individuals at risk for underuse.

## Introduction

Besides cardiovascular diseases, cancer is one of the leading causes of death 1. Since several types of cancer are more common in older adults, the growing number of individuals in old age will most likely increase the prevalence of cancer 2. Nevertheless, the survival rates of patients suffering from cancer increased in the past years, often attributed to advances in cancer therapy and prevention strategies 3. While primary prevention aims at reducing the incidence of a disorder, secondary prevention aims at *early* detection and treatment of diseases. A well‐known secondary prevention strategy is cancer screening including Papanicolaou smear, mammography screening, or colorectal cancer screening. In Germany, cancer screenings are voluntary, but numerous cancer screenings are paid by statutory health insurances. Internationally, the World Health Organization has defined criteria and created guidelines for screenings 4. In line with these guidelines, in Germany, screenings are only paid by statutory health insurances when their efficacy was confirmed.

Like in many other countries, the use of cancer screenings is actively promoted by the government in Germany 5. Nevertheless, these screenings are used infrequently. For example, 62.9% of the individuals aged 40—85 years in Germany reported to regularly use cancer screenings in the past years, with marked sex differences (women: 72.6%, men: 52.6%) 6. Besides sex, the use of cancer screening is positively associated with age 7, 8. Moreover, the use of cancer screening is positively associated with education 8. Furthermore, it has been demonstrated that there is an association between need factors (self‐rated health and morbidity) and use of cancer screening 9, 10. Many studies focused on socioeconomic predictors for preventive screenings 11, 12 or used the Andersen and Newman theoretical framework 13.

Beyond these associations, several studies used the health‐belief model to examine the association between psychosocial variables and the use of cancer screening. For example, it was found that beliefs in the efficacy of screenings (perceived benefit) or optimism about cancer were associated with the use of cancer screening 14, 15, 16, 17. However, these studies mainly focused on *illness‐specific* psychosocial factors. Thus, studies are missing investigating the association between more *general* psychosocial factors (such as general self‐efficacy, self‐esteem, perceived social exclusion, affective well‐being, or general optimism). However, it appears plausible that these general psychosocial factors are strongly related with health‐related or health‐promotion behavior including eating a healthy diet, exercising regularly, moderate alcohol intake, getting sufficient rest, and, more generally, health responsibility behaviors 18, 19, 20. Hence, we assume that general psychosocial factors are also associated with the use of cancer screening.

Consequently, using a large, population‐based sample of community‐dwelling individuals aged 40 and over, we aimed at examining the association between general well‐established psychosocial factors (loneliness, cognitive well‐being (CWB), affective well‐being (AWB, negative (NA) and positive affect (PA)), optimism, self‐efficacy, self‐esteem, self‐regulation, perceived autonomy, perceived stress, and perceived social exclusion) and the use of cancer screening. Knowing general psychosocial factors that are associated with the use of cancer screening might be fruitful to address individuals at risk for underuse 21.

In order to get a general idea about these general psychosocial factors used in our study, these factors are shortly defined as follows 22: Loneliness is the state that an individual's social network is smaller than desired. While CWB (life satisfaction) refers to the cognitive evaluation of the life as a whole, affective well‐being refers to PA (emotions such as joy) and NA (emotions such as anger). Optimism is the belief that rather good things than bad things will occur. Self‐efficacy is defined as the belief in one's abilities to change. Self‐regulation is characterized by controlled processing (based on the theory of selection, optimization, and compensation (SOC)). For example, individuals scoring high in self‐regulation are more willing to delay short‐term satisfaction to meet long‐term needs. Perceived autonomy in old age is mainly defined as the experience of choice. Perceived stress is characterized as the degree to which situations in one's life are viewed as stressful. Perceived social exclusion is the feeling that one does not belong to the society.

## Methods

### Sample

In our study, data were gathered from the fifth wave (2014) of the German Ageing Survey (DEAS), funded by the Federal Ministry for Family Affairs, Senior Citizens, Women and Youth (BMFSFJ). Starting in 1996, it is a population‐based survey among individuals aged 40 and above. Data were collected about occupational status, life contexts, social ties, income, health, general psychosocial factors, and so forth. In total, 7673 individuals filled out the drop‐off questionnaire in 2014 and provided data on cancer screening. The response rate for participants who had already taken part before was 61% in 2014, the response rate for first‐time participants was 25%. The response rate of this survey is comparable to other survey studies conducted in Germany. More details about this survey were provided elsewhere 23. Since we were interested in a comprehensive description of the German population in 2014, we used the fifth wave of the DEAS that provided data on perceived social exclusion, perceived stress, perceived autonomy and self‐regulation. Prior to the interview, written informed consent was given.

Please note that an ethical statement for this study was not necessary because criteria for the need of an ethical statement were not met (risk for the respondents, lack of information about the aims of the study, examination of patients). The principles outlined in the Helsinki Declaration were followed.

### Dependent variable

To quantify the use of cancer screening, individuals were asked whether they regularly underwent early cancer screening in the past years (yes; no).

### Independent variables: Psychosocial factors

NA and PA were quantified using the positive and negative affect schedule (PANAS) 24 which has excellent psychometric properties 25. The PANAS consists of 10 items (in each case from 1 =  “very slightly or not at all” to 5 =  “extremely”). An index (1–5) was created for (1) PA and (2) NA by averaging the score of the corresponding items, with higher values representing higher PA as well as higher NA. In our study, Cronbach's Alpha was 0.87 for the PANAS.

CWB was measured by the well‐validated 26 Satisfaction with Life Scale (SWLS) 27. The scale comprises five items about their satisfaction with life as a whole, each ranging from 1 =  “strongly agree” to 5 =  “strongly disagree” (index score from 1 to 5, with higher values reflecting higher CWB). Cronbach's Alpha was 0.86 in our study.

Loneliness was quantified using a short version 28 (six items) of the well‐validated De Jong Gierveld Loneliness Scale 29 (11 items). The short version distinguishing between emotional and social loneliness (both with 3 items) has proven to be valid and reliable 30. Items range between 1 (strongly agree) and 4 (strongly disagree). An index value (1–4) was created by averaging the items. The higher the values, the higher the loneliness (Cronbach's Alpha = 0.83).

Optimism was assessed using a validated scale (five items, ranging from 1 = “strongly agree” to 4 = “strongly disagree”) developed by Brandtstädter and Wentura 31. The index score also ranges from 1 to 4, with higher values reflecting high optimism (Cronbach's Alpha = 0.84).

Perceived social exclusion was measured using a scale (four items, ranging from 1 to 5) developed by Bude and Lantermann 32. The higher the index score (1–5), the higher perceived social exclusion. Cronbach's Alpha was 0.88.

Perceived autonomy was quantified using a scale developed by Schwarzer 33, consisting of four items (index score from 1 to 5). High values of the index correspond with high perceived autonomy (Cronbach's Alpha = 0.81).

A measure of perceived stress developed by Cohen et al. 34 was used. It consists of four items, with higher values reflecting higher self‐rated stress. The index score ranges from 1 to 5 (high values indicate high subjective stress). Cronbach's Alpha was 0.70.

Self‐regulation was measured using a scale by Ziegelmann and Lippke 35 which was based on a scale originally developed by Freund and Baltes 36. Self‐regulation is based on the theory of SOC. The validated scale covers four items (higher values reflect higher self‐regulation; index score from 1 to 5; Cronbach's Alpha was 0.78).

The validated Rosenberg scale 37, consisting of 10 items (from 1 =  “strongly agree” to 4 =  “strongly disagree”), was used to assess self‐esteem. By averaging the items, an index score (1–4) was generated. Higher values correspond to a greater level of self‐esteem (Cronbach's Alpha was 0.84).

According to Schwarzer and Jerusalem 38, 39, self‐efficacy was quantified (five items, each from 1 =  “strongly agree” to 4 =  “strongly disagree”). An index score (1–4) was computed by averaging the items. High values reflect high self‐efficacy. Cronbach's alpha was 0.75.

### Independent variables: Potential confounders

Furthermore, we used numerous potential confounders in our regression models including age, marital status (married, living together with spouse, others (married, living separated from spouse; divorced; widowed; never married), and employment status (working; retired; not employed). In addition, monthly net equivalence income in Euro (new OECD scale) was used. The region was considered distinguishing between West and East Germany, the latter defined by the area of the former German Democratic Republic. Moreover, subjective health as well as morbidity was included. A self‐rated scale (from 1 =  “very good” to 5 =  “very bad”) was used to quantify subjective health. The number of physical diseases (adapted from the Charlson Comorbidity Index 40) was used to assess morbidity.

In addition, several lifestyle factors were included. The self‐reported Body Mass Index (BMI) was used. Furthermore, alcohol consumption was measured as days with alcohol consumption (e.g., beer, wine, sparkling wine, spirits, long drinks) with “daily,” “several times a week,” “once a week,” “1 to 3 times a month,” “less often,” and “never.” It was dichotomized (daily alcohol consumption (“daily”) vs. less than daily alcohol consumption (otherwise)). Moreover, the current smoking status (yes, daily; yes, sometimes; not anymore; never been a smoker) was considered. Furthermore, physical activities were quantified by asking “How often do you do sports such as hiking, soccer, gymnastics, or swimming?” (daily; several times a week; once a week; 1–3 times a month; less often). It was dichotomized (at least once a week (daily; several times a week; once a week) vs. less than once a week (otherwise)).

Categorical variables were dichotomized (e.g., smoking: yes/no; binary variable) in main analysis for reasons of clarity. In sensitivity analysis, categorical variables with k categories (e.g., frequency of alcohol consumption, smoking status) were transformed into k‐1 dummy variables before being entered into the regression model. We also controlled for social strata (lower class; lower middle class; middle class; upper middle class; upper class 41) in further sensitivity analysis.

### Statistical analysis

Pairwise Pearson correlations were computed to gain some insight into the relationships between the variables. Furthermore, multiple logistic regressions were used to investigate the association between regressors and the regular use of cancer screening in the past years. The level of significance was fixed at 5%. Statistical analyses were conducted using Stata 14.0 (StataCorp, College Station, Texas).

## Results

### Sample characteristics

Sample characteristics are displayed in Table 1. In the sample, mean age was 64.3 years (±11.2 years; 40–95). Fifty‐one percent of the individuals were females, 54.1% of the individuals were retired, 67.2% lived in West Germany, and 70.2% of the individuals were married, living together with spouse. The mean monthly net equivalence income in Euro was 1948.9 €. The percentage of the individuals who exercised at least once a week was 54.2%. The mean BMI was 26.9 (±4.6). Twelve percent of the individuals had daily alcohol intake, and 18.0% of the individuals were current smokers. The mean number of physical diseases was 2.6 (±1.9) and the mean self‐rated health was 2.5 (±0.8). A total of 65.6% of the individuals regularly underwent early cancer screening in the past years. Moreover, the general psychosocial factors (mean and SD) are displayed in Table 1.

**Table 1:** Descriptive statistics (*n* = 7673; Germany, in 2014)

Variable	*N* (%)/Mean (SD); Range
Female (Ref.: Male): *N* (%)	3916 (51.0)
Age: Mean (SD); Range	64.3 (11.2); 40–95
Employment status^a^: *N* (%)	
Working	2837 (37.0)
Retired	4149 (54.1)
Not employed	684 (8.9)
Married, living together with spouse^b^: *N* (%)	5374 (70.2)
Monthly net equivalence income in Euro^c^: Mean (SD); Range	1948.9 (1379.6)
East Germany (Ref.: West Germany): *N* (%)	2516 (32.8)
Sports at least once a week (Ref.: Sports less than once a week)^d^: *N* (%)	4155 (54.2)
Body Mass Index (BMI)^e^: Mean (SD); Range	26.9 (4.6); 13.2–60.9
Current smoker (Ref.: No)^f^: *N* (%)	1366 (18.0)
Daily alcohol consumption (Ref.: Less than daily alcohol consumption)^g^: *N* (%)	919 (12.0)
Number of physical diseases^h^: Mean (SD); Range	2.6 (1.9); 0–11
Self‐rated health^i^: Mean (SD); Range	2.5 (0.8); 1–5
Loneliness^j^ ^28^: Mean (SD); Range	1.8 (0.5); 1–4
Cognitive well‐being^k^ [SWLS 27): Mean (SD); Range	3.8 (0.7); 1–5
Positive affect^l^ (PANAS 24): Mean (SD); Range	3.6 (0.5); 1–5
Negative affect^m^ (PANAS 24): Mean (SD); Range	2.1 (0.5); 1–5
Optimism^n^ ^31^: Mean (SD); Range	3.0 (0.6); 1–4
Self‐efficacy^o^ ^38^: Mean (SD); Range	3.1 (0.4); 1–4
Self‐esteem^p^ ^37^: Mean (SD); Range	3.4 (0.4); 1.2–4
Self‐regulation^q^ ^36^: Mean (SD); Range	4.0 (0.5); 2–5
Perceived autonomy^r^ ^33^: Mean (SD); Range	4.5 (0.5); 2–5
Perceived stress^s^ ^34^: Mean (SD); Range	2.4 (0.7); 1–5
Perceived social exclusion^t^ ^32^: Mean (SD); Range	2.6 (0.6); 2–5
Use of cancer screening (Yes): *N* (%)	5034 (65.6)

a 3 missing values.

b 16 missing values.

c 422 missing values.

d 1 missing value.

e 135 missing values.

f 61 missing values.

g 16 missing values.

h 115 missing values.

i 7 missing values.

j 112 missing values.

k 67 missing values.

l 75 missing values.

m 75 missing values.

n 25 missing values.

o 26 missing values.

p 9 missing values.

q 151 missing values.

r 59 missing values.

s 109 missing values.

t 92 missing values.

### Correlations

Pairwise Pearson correlations are depicted in Table 2. The use of cancer screening was positively associated with being female (*r* = 0.14, *P* < 0.001), married, living together with spouse (*r* = 0.10, *P* < 0.001), exercising at least once a week (*r* = 0.12, *P* < 0.001), and nonsmoking (*r* = −0.14, *P* < 0.001). However, the outcome variable was not significantly associated with age, daily alcohol consumption, employment status, income, region, BMI, number of physical diseases, and self‐rated health.

**Table 2:** Pairwise correlations (with Bonferroni correction for multiple comparisons). (Germany, in 2014)

	Use of cancer screening (Ref.: no)	Female (Ref.: Male)	Age	Retired (Ref.: Working)	Not employed	Married, living together with spouse	Monthly net equivalence income in Euro	East Germany (Ref.: West Germany)	Sports at least once a week (Ref.: Sports less than once a week)	Body Mass Index (BMI)	Current smoker (Ref.: No)	Daily alcohol consumption (Ref.: Less than daily alcohol consumption)	Number of physical diseases	Self‐rated health	Loneliness	Cognitive well‐being	Positive affect	Negative affect	Optimism	Self‐efficacy	Self‐esteem	Self‐regulation	Perceived autonomy	Perceived stress	Perceived social exclusion
Use of cancer screening (Ref.: no)	1.00																								
Female (Ref.: Male)	0.16***	1.00																							
Age	−0.01	−0.09***	1.00																						
Retired (Ref.: Working)	−0.01	−0.09***	0.78***	1.00																					
Not employed	−0.02	0.11***	−0.13***	−0.34***	1.00																				
Married, living together with spouse	0.10***	−0.14***	−0.02	−0.02	−0.02	1.00																			
Monthly net equivalence income in Euro	0.04	−0.05*	−0.09***	−0.13***	−0.07***	0.09***	1.00																		
East Germany (Ref.: West Germany)	0.01	0.02	0.02	0.03	−0.01	−0.02	−0.19***	1.00																	
Sports at least once a week (Ref.: Sports less than once a week)	0.12***	0.10***	−0.07***	−0.03	−0.03	0.04	0.15***	−0.11***	1.00																
Body Mass Index (BMI)	−0.02	−0.10***	0.03	0.05**	0.02	0.02	−0.11***	0.09***	−0.18***	1.00															
Current smoker (Ref.: No)	−0.09***	−0.03	−0.25***	−0.20***	0.10***	−0.11***	−0.04	−0.01	−0.15***	−0.03	1.00														
Daily alcohol consumption (Ref.: Less than daily alcohol consumption)	−0.04+	−0.19***	0.08***	0.08***	−0.04*	0.04	0.07***	−0.04	−0.02	−0.04	0.02	1.00													
Number of physical diseases	0.02	−0.04+	0.35***	0.29***	−0.01	−0.06***	−0.14***	0.03	−0.13***	0.21***	−0.06***	0.02	1.00												
Self‐rated health	−0.02	−0.02	0.13***	0.13***	0.04	−0.04	−0.15***	0.06***	−0.20***	0.22***	0.04	−0.01	0.43***	1.00											
Loneliness 28	−0.08***	−0.08***	−0.06**	−0.02	0.05*	−0.11***	−0.09***	−0.08***	−0.06***	0.04	0.06***	0.01	0.17***	0.20***	1.00										
Cognitive well‐being (SWLS 27)	0.09***	0.03	0.11***	0.06***	−0.12***	0.19***	0.18***	−0.01	0.10***	−0.06***	−0.12***	0.01	−0.20***	−0.32***	−0.49***	1.00									
Positive affect (PANAS 24)	0.09***	0.06***	−0.11***	−0.09***	−0.04	0.05*	0.15***	−0.01	0.16***	−0.08***	−0.01	−0.00	−0.24***	−0.33***	−0.40***	0.47***	1.00								
Negative affect (PANAS 24)	0.02	0.14***	−0.17***	−0.12***	0.06***	−0.03	−0.07***	−0.09***	−0.01	−0.00	0.06**	−0.01	0.18***	0.22***	0.43***	−0.41***	−0.31***	1.00							
Optimism 31	0.06***	0.01	−0.13***	−0.12***	−0.05**	0.09***	0.16***	−0.04+	0.13***	−0.06***	−0.04	−0.01	−0.30***	−0.39***	−0.45***	0.61***	0.57***	−0.39***	1.00						
Self‐efficacy 38	0.03	−0.01	−0.06***	−0.06***	−0.04	0.03	0.12***	−0.00	0.05*	−0.02	0.00	−0.00	−0.21***	−0.25***	−0.36***	0.48***	0.53***	−0.38***	0.58***	1.00					
Self‐esteem 37	0.08***	0.04	−0.04	−0.05**	−0.07***	0.07***	0.15***	0.01	0.10***	−0.08***	−0.04	−0.00	−0.25***	−0.29***	−0.53***	0.54***	0.60***	−0.52***	0.61***	0.61***	1.00				
Self‐regulation 36	0.04	0.03	0.04	0.04+	−0.04+	−0.00	0.03	0.08***	0.04	−0.00	−0.03	−0.02	−0.11***	−0.13***	−0.29***	0.37***	0.42***	−0.22***	0.38***	0.47***	0.39***	1.00			
Perceived autonomy 33	0.04	0.13***	−0.08***	−0.07***	−0.00	−0.13***	0.09***	0.01	0.08***	−0.07***	0.02	−0.02	−0.20***	−0.23***	−0.21***	0.25***	0.31***	−0.23***	0.31***	0.40***	0.37***	0.32***	1.00		
Perceived stress 34	−0.04+	0.06***	−0.03	−0.03	0.05*	−0.08***	−0.16***	−0.00	−0.12***	0.07***	0.07***	−0.04+	0.24***	0.33***	0.45***	−0.45***	−0.50***	0.51***	−0.52***	−0.45***	−0.52***	−0.29***	−0.28***	1.00	
Perceived social exclusion 32	−0.05**	0.02	0.02	0.04	0.10***	−0.09***	−0.17***	0.05**	−0.10***	0.06***	0.04	−0.03	0.22***	0.24***	0.50***	−0.42***	−0.41***	0.41***	−0.48***	−0.39***	−0.60***	−0.22***	−0.26***	0.42***	1.00
Observations		6961																							

***P < 0.001, **P < 0.01, *P < 0.05, ^+^ *P* < 0.10.

As for general psychosocial variables, the use of cancer screening was positively associated with less loneliness (*r* = −0.08, *P* < 0.001), CWB (*r* = 0.09, *P* < 0.001), PA (*r* = 0.09, *P* < 0.001), optimism (*r* = 0.06, *P* < 0.001), self‐esteem (*r* = 0.08, *P* < 0.001), and decreased perceived social exclusion (*r* = −0.05, *P* < 0.01), whereas it was not significantly related to NA, self‐efficacy, self‐regulation, perceived autonomy, and perceived stress.

### Regression analysis

Results of multiple logistic regressions are displayed in Table 3. Adjusting for potential confounders, the regressions showed that the use of cancer screening is positively associated with decreased loneliness [OR: 0.81 (0.73–0.89)], CWB [OR: 1.21 (1.12–1.31)], optimism [OR: 1.23 (1.11–1.36)], self‐efficacy [OR: 1.19 (1.05–1.34)], self‐esteem [OR: 1.43 (1.25–1.64)], self‐regulation [OR: 1.16 (1.04–1.28)], perceived autonomy [OR: 1.16 (1.05–1.28)], decreased perceived stress [OR: 0.86 (0.79–0.94)], decreased perceived social exclusion [OR: 0.84 (0.77–0.93)], and PA [OR: 1.38 (1.24–1.53)], but not with NA.

**Table 3:** Predictors of the use of cancer screening (0 =  no; 1 =  yes). Results of multiple logistic regressions. (Germany, in 2014)

Variables	(1)	(2)	(3)	(4)	(5)	(6)	(7)	(8)	(9)	(10)	(11)
	Cancer screening	Cancer screening	Cancer screening	Cancer screening	Cancer screening	Cancer screening	Cancer screening	Cancer screening	Cancer screening	Cancer screening	Cancer screening
Female (Ref.: Male)	2.087***	2.091***	2.102***	2.147***	2.126***	2.118***	2.097***	2.114***	2.094***	2.160***	2.133***
	(1.871–2.329)	(1.875–2.332)	(1.885–2.343)	(1.924–2.395)	(1.908–2.370)	(1.900–2.360)	(1.882–2.337)	(1.895–2.358)	(1.878–2.334)	(1.937–2.409)	(1.913–2.378)
Age	0.991*	0.990*	0.994	0.993+	0.993+	0.993+	0.991*	0.991*	0.992*	0.992*	0.992*
	(0.983–0.999)	(0.982–0.998)	(0.986–1.001)	(0.985–1.000)	(0.985–1.000)	(0.985–1.000)	(0.984–0.999)	(0.983–0.999)	(0.984–1.000)	(0.984–1.000)	(0.984–0.999)
Retired (Ref.: Working)	1.082	1.083	1.055	1.063	1.070	1.055	1.075	1.060	1.064	1.053	1.084
	(0.904–1.295)	(0.905–1.296)	(0.882–1.263)	(0.888–1.271)	(0.895–1.279)	(0.882–1.261)	(0.899–1.285)	(0.886–1.269)	(0.890–1.272)	(0.880–1.260)	(0.906–1.298)
Not employed	0.851	0.866	0.839+	0.829+	0.848	0.836+	0.860	0.836+	0.837+	0.837+	0.866
	(0.696–1.042)	(0.708–1.059)	(0.686–1.025)	(0.679–1.013)	(0.694–1.036)	(0.685–1.022)	(0.703–1.051)	(0.683–1.022)	(0.685–1.023)	(0.684–1.024)	(0.708–1.060)
Married, living together with spouse	1.688***	1.653***	1.722***	1.740***	1.717***	1.735***	1.712***	1.741***	1.779***	1.716***	1.723***
	(1.506–1.892)	(1.474–1.854)	(1.538–1.929)	(1.555–1.948)	(1.534–1.922)	(1.550–1.941)	(1.529–1.916)	(1.554–1.951)	(1.588–1.992)	(1.532–1.922)	(1.539–1.930)
Monthly net equivalence income in Euro	1.000*	1.000+	1.000+	1.000*	1.000*	1.000*	1.000+	1.000*	1.000*	1.000*	1.000*
	(1.000–1.000)	(1.000–1.000)	(1.000–1.000)	(1.000–1.000)	(1.000–1.000)	(1.000–1.000)	(1.000–1.000)	(1.000–1.000)	(1.000–1.000)	(1.000–1.000)	(1.000–1.000)
East Germany (Ref.: West Germany)	1.121*	1.132*	1.128*	1.134*	1.141*	1.141*	1.130*	1.142*	1.133*	1.136*	1.148*
	(1.001–1.256)	(1.012–1.266)	(1.008–1.262)	(1.013–1.269)	(1.020–1.276)	(1.021–1.276)	(1.011–1.264)	(1.020–1.279)	(1.014–1.267)	(1.015–1.272)	(1.026–1.284)
Sports at least once a week (Ref.: Sports less than once a week)	1.519***	1.499***	1.464***	1.501***	1.492***	1.505***	1.496***	1.509***	1.489***	1.507***	1.494***
	(1.363–1.693)	(1.345–1.670)	(1.313–1.632)	(1.347–1.672)	(1.340–1.663)	(1.351–1.677)	(1.343–1.666)	(1.354–1.683)	(1.337–1.659)	(1.352–1.680)	(1.341–1.665)
Body Mass Index (BMI)	0.997	0.997	0.998	0.999	0.997	0.997	0.997	0.996	0.998	0.997	0.997
	(0.985–1.009)	(0.985–1.009)	(0.986–1.010)	(0.987–1.011)	(0.985–1.009)	(0.985–1.009)	(0.985–1.009)	(0.984–1.008)	(0.986–1.010)	(0.985–1.009)	(0.985–1.009)
Current smoker (Ref.: No)	0.721***	0.733***	0.721***	0.727***	0.726***	0.717***	0.720***	0.719***	0.712***	0.720***	0.728***
	(0.628–0.827)	(0.639–0.842)	(0.628–0.828)	(0.634–0.835)	(0.632–0.833)	(0.625–0.823)	(0.628–0.827)	(0.626–0.826)	(0.621–0.817)	(0.627–0.826)	(0.635–0.836)
Daily alcohol consumption (Ref.: Less than daily alcohol consumption)	0.902	0.897	0.897	0.901	0.908	0.907	0.911	0.897	0.909	0.894	0.899
	(0.772–1.053)	(0.768–1.047)	(0.768–1.048)	(0.772–1.053)	(0.778–1.060)	(0.777–1.059)	(0.780–1.064)	(0.768–1.048)	(0.778–1.061)	(0.765–1.044)	(0.769–1.050)
Number of physical diseases	1.086***	1.084***	1.084***	1.077***	1.084***	1.079***	1.088***	1.076***	1.080***	1.086***	1.084***
	(1.050–1.122)	(1.049–1.121)	(1.049–1.120)	(1.042–1.114)	(1.049–1.121)	(1.044–1.115)	(1.053–1.125)	(1.041–1.112)	(1.045–1.115)	(1.051–1.123)	(1.049–1.121)
Self‐rated health	1.002	1.032	1.036	0.987	1.025	0.999	1.020	0.998	0.996	1.014	1.005
	(0.934–1.076)	(0.960–1.110)	(0.964–1.114)	(0.920–1.059)	(0.953–1.102)	(0.931–1.072)	(0.950–1.095)	(0.930–1.070)	(0.928–1.069)	(0.943–1.090)	(0.936–1.078)
Loneliness 28	0.805***										
	(0.729–0.889)										
Cognitive well‐being (SWLS 27)		1.208***									
		(1.117–1.306)									
Positive affect (PANAS 24)			1.377***								
			(1.239–1.531)								
Negative affect (PANAS 24)				0.961							
				(0.864–1.068)							
Optimism 31					1.229***						
					(1.109–1.362)						
Self‐efficacy 38						1.186**					
						(1.049–1.341)					
Self‐esteem 37							1.429***				
							(1.250–1.635)				
Self‐regulation 36								1.157**			
								(1.044–1.283)			
Perceived autonomy 33									1.159**		
									(1.045–1.284)		
Perceived stress 34										0.864***	
										(0.793–0.941)	
Perceived social exclusion 32											0.844***
											(0.770–0.925)
Constant	1.624	0.543+	0.273***	1.043	0.503*	0.593	0.302**	0.639	0.517+	1.395	1.575
	(0.858–3.072)	(0.286–1.030)	(0.133–0.562)	(0.541–2.009)	(0.254–0.995)	(0.293–1.202)	(0.143–0.636)	(0.313–1.303)	(0.240–1.111)	(0.746–2.611)	(0.826–3.004)
Observations	6875	6915	6909	6908	6949	6946	6961	6842	6947	6877	6900
Pseudo *R*²	0.051	0.050	0.052	0.048	0.050	0.048	0.051	0.049	0.049	0.050	0.049

Odds ratios were reported. 95% confidence intervals in parentheses. ***P < 0.001, **P < 0.01, *P < 0.05, ^+^ *P* < 0.10.

Furthermore, while the outcome variable was positively associated with being female, age (in some model specifications), income (in some model specifications), East Germany, exercising at least once a week, nonsmoking, and the number of physical diseases, it was not associated with employment status, BMI, alcohol consumption, and self‐rated health.

In sensitivity analysis, categorical variables (e.g., smoking status) were transformed into k‐1 dummy variables before being entered into the regression model. However, the association between psychosocial factors and the use of cancer screening remained almost the same in terms of significance and effect sizes (results not shown, but available upon request). In further sensitivity analysis, we also controlled for social strata. Again, results remained virtually the same. However, self‐efficacy was only marginally significant [OR: 1.15 (0.98–1.33), *P* = 0.08].

## Discussion

### Main findings

Using a large, population‐based sample of community‐dwelling individuals aged 40 and above in Germany, this study aimed at examining the association between the use of cancer screening and a number of general psychosocial factors. Multiple logistic regressions revealed that the use of cancer screening is positively associated with decreased loneliness, CWB, optimism, self‐efficacy, self‐esteem, self‐regulation, perceived autonomy, decreased perceived stress, decreased perceived social exclusion, and PA, whereas it is not associated with NA.

### Previous research

Our findings regarding the association between *general* well‐established psychosocial factors and the use of cancer screening extend previous knowledge about an association between *illness‐specific* beliefs in the efficacy of screenings and use of cancer screening. Thus, based on the health‐belief model most of the previous studies discussed below focused on illness‐specific psychosocial factors and the use of cancer screening, often in very specific samples. Our data indicate that it is worth investigating the association between general psychosocial factors and the use of cancer screening because these factors are associated with our outcome measures.

In total, it is difficult to compare previous studies with our findings since most of the previous studies focused on illness‐specific rather than more general psychosocial factors which is worth repeating here. Thus, first, psychosocial factors analyzed in our study will be discussed in relation to existing studies investigating more general psychosocial factors. Second, psychosocial factors will be discussed in relation to existing studies in which more illness‐specific psychosocial factors were examined. Third, psychosocial factors, where there is very little evidence at all, will be discussed.

As for loneliness, some studies investigating the association between social ties and the use of cancer screening exist. For example, Messina et al. 42 found that repeated breast cancer screening was positively associated with emotional/informational support and positive social interactions in older women. In addition, Kinney et al. 43 showed that social ties were positively associated with the use of colorectal cancer screening in older women. In total, our findings regarding loneliness confirm previous studies undertaken in the United States.

In our study, the use of cancer screening was positively associated with lower perceived stress. This might be explained by the fact that stress is negatively associated with health‐promotion behavior 19. Consequently, the lower the perceived stress, the higher the probability of using cancer screenings. However, Wardle et al. 44 reported that perceived stress was not significantly associated with the intention to undertake bowel cancer screening. Another study also found that stress coping with the environment was not significantly associated with prostate cancer screening in African American men 45. The findings of these studies might be explained by the fact that stress is positively associated with neuroticism 46. This factor is in turn positively related to higher health care use 47. Consequently, these negative factors might counterbalance other health‐related behaviors mentioned above.

Hay et al. 48 conducted a meta‐analysis regarding the association between worry about breast cancer and screening behavior. They found that nearly all studies reported a positive relationship between cancer worry and screening behavior. We found no association between the more general NA and use of cancer screening. This might be explained as follows: It has been demonstrated that NA is strongly associated with depression as well as anxiety 25. This might be in accordance with another study which found a nonsignificant association between general anxiety and mammography use in women aged 18–74 years 18. In contrast, we obtained a positive association between PA and the use of cancer screening which might be explained by the link between PA and health‐promotion behavior 49. However, more research is required to clarify the different association between the use of cancer screening and PA as well as NA.

Self‐regulation was positively associated with the use of cancer screening in our study. This is also in line with Consedine et al. 50 investigating the association between emotional factors and breast cancer screening in women. They found that screening was positively associated with self‐regulation. A possible explanation might be that the higher the self‐regulation, the higher the willingness to postpone satisfaction of short‐term needs to satisfy long‐term needs. Consequently, individuals scoring high in self‐regulation might be more willing to undergo regularly cancer screening to satisfy the long‐term goal of staying healthy. Hence, they might be more willing to deal with short‐term negative circumstances of cancer screenings (e.g., “sacrificing” leisure time or unpleasant screening procedure) to achieve long‐term goals.

Regarding self‐efficacy, several studies 51, 52, 53 have shown that belief in the efficacy of screening is associated with cancer screenings. In our study, it was found that general self‐efficacy was associated with the use of cancer screening. This association might be explained by the fact that individuals scoring high in self‐efficacy believe in their own abilities to reach goals (e.g., quit smoking, or do breast self‐examination). Thus, it is highly plausible that self‐efficacy is strongly associated with the use of cancer screening.

Only a few studies have found that optimism was positively associated with cancer prevention 15, 54. For example, Wardle et al. 54 found that optimism was positively associated with interest in participating in bowel cancer screening in older adults. A positive association was also found for the relation between optimism about cancer prevention and colorectal as well as prostate cancer 15. Extending previous studies, our study found that general optimism was associated with the use of cancer screening. This might be mainly explained by the fact that optimism is related with protective health behaviors 55 such as eating a healthy diet 56. It was suggested that optimistic individuals are more likely to adopt as well as maintain positive health behaviors 20, 57.

Yet, there is little evidence that self‐esteem is associated with the use of cancer screening. For example, it was found that women with high levels of self‐esteem have more positive health beliefs on breast cancer screening 58. Our findings (positive association between use of cancer screening and self‐esteem) might be explained by the fact low self‐esteem is associated with a negative body image 59. This, in turn is linked with a decreased use of cancer screening 60.

We found a positive association between CWB and the use of cancer screening. Thus far, studies examining the association between CWB and the use of cancer screening are missing. A possible explanation might be that CWB is strongly associated with health‐related behavior 61 which is in turn related to the use of cancer screening.

Studies analyzing the relation between perceived social exclusion and use of cancer screening are missing. However, it was shown that ethnic factors are associated with cervical cancer screening in a systematic review 62. These ethnic factors might be associated with stigmatization by physicians or feelings of perceived social exclusion. Consequently, these findings might be in accordance with our findings showing a positive association between decreased perceived social exclusion and the use of cancer screening.

In our study, perceived autonomy was positively associated with the use of cancer screening. To the best of our knowledge, no study has explicitly focused on the association between autonomy and the use of cancer screening. Since higher perceived autonomy is associated with greater perceived freedom and higher physical health 63, it appears plausible that autonomy is positively associated with the use of cancer screening. Moreover, it is worth noting that our findings regarding our control variables (e.g., sex, smoking and drinking behavior as well as self‐rated health and morbidity) are mostly in accordance with previous studies 64, 65, 66.

### Strengths and limitations

This is the first study focusing on the association between numerous general psychosocial factors and the use of cancer screening. Data were gathered from a large, representative study of community‐dwelling individuals aged 40 and over. Well‐established and widely used general psychosocial constructs were applied. Furthermore, numerous important independent variables (e.g., sociodemographic variables, need factors as well as lifestyle variables) were included in regression models.

Nevertheless, this study has several limitations. This is a cross‐sectional study, thus limiting our ability to determine cause‐and‐effect relations. Longitudinal studies are needed in order to measure the influence of general psychosocial factors on the use of cancer screening. As these general psychosocial factors are potentially modifiable, this is important to develop interventional strategies. Furthermore, instrumental variable approaches can be used to deal with endogeneity issues (e.g., simultaneity bias). However, these approaches rest on very strong assumptions. When these strong assumptions are not fulfilled (e.g., weak instruments), estimates are heavily biased. Therefore, instrumental variable approaches were not used in this study. Moreover, we cannot distinguish between the different types of cancer screenings for reasons of data availability in the German Ageing Survey. However, our findings provide some insights into the association between numerous general psychosocial factors and the use of cancer screening *in general* (based on a large, nationally representative sample with established measures for the psychosocial variables). Nevertheless, future studies are required to disentangle the association between general psychosocial factors and different types of cancer screenings such as colorectal cancer screening or breast cancer screening. It might be the case that the relationship with psychosocial factors investigated in this study depends on the type of screening.

## Conclusions

Even though cancer screenings are voluntary, they are often actively promoted by the governments of countries. However, many screenings are used infrequently. To facilitate addressing this potential problem, it is important to address persons at risk for underuse of cancer screening. Knowing general psychosocial factors that are associated with the use of cancer screening might be fruitful to address these individuals.

## Conflict of Interest

The authors declare that they have no competing interests.
